# The *K_a_ /K_s_* and *π_a_ /π_s_* Ratios under Different Models of Gametophytic and Sporophytic Selection

**DOI:** 10.1093/gbe/evad151

**Published:** 2023-08-10

**Authors:** Ling-Ling Li, Yu Xiao, Xi Wang, Zi-Han He, Yan-Wen Lv, Xin-Sheng Hu

**Affiliations:** College of Forestry and Landscape Architecture, South China Agricultural University, Guangzhou 510642, China; Guangdong Key Laboratory for Innovative Development and Utilization of Forest Plant Germplasm, South China Agricultural University, Guangzhou 510642, China; College of Forestry and Landscape Architecture, South China Agricultural University, Guangzhou 510642, China; Guangdong Key Laboratory for Innovative Development and Utilization of Forest Plant Germplasm, South China Agricultural University, Guangzhou 510642, China; College of Forestry and Landscape Architecture, South China Agricultural University, Guangzhou 510642, China; Guangdong Key Laboratory for Innovative Development and Utilization of Forest Plant Germplasm, South China Agricultural University, Guangzhou 510642, China; College of Forestry and Landscape Architecture, South China Agricultural University, Guangzhou 510642, China; Guangdong Key Laboratory for Innovative Development and Utilization of Forest Plant Germplasm, South China Agricultural University, Guangzhou 510642, China; College of Forestry and Landscape Architecture, South China Agricultural University, Guangzhou 510642, China; Guangdong Key Laboratory for Innovative Development and Utilization of Forest Plant Germplasm, South China Agricultural University, Guangzhou 510642, China; College of Forestry and Landscape Architecture, South China Agricultural University, Guangzhou 510642, China; Guangdong Key Laboratory for Innovative Development and Utilization of Forest Plant Germplasm, South China Agricultural University, Guangzhou 510642, China

**Keywords:** mating system, evolutionary rate, alternation of generations, positive selection, purifying selection

## Abstract

Alternation of generations in plant life cycle provides a biological basis for natural selection occurring in either the gametophyte or the sporophyte phase or in both. Divergent biphasic selection could yield distinct evolutionary rates for phase-specific or pleiotropic genes. Here, we analyze models that deal with antagonistic and synergistic selection between alternative generations in terms of the ratio of nonsynonymous to synonymous divergence (*K_a_/K_s_*). Effects of biphasic selection are opposite under antagonistic selection but cumulative under synergistic selection for pleiotropic genes. Under the additive and comparable strengths of biphasic allelic selection, the absolute *K_a_/K_s_* for the gametophyte gene is equal to in outcrossing but smaller than, in a mixed mating system, that for the sporophyte gene under antagonistic selection. The same pattern is predicted for *K_a_/K_s_* under synergistic selection. Selfing reduces efficacy of gametophytic selection. Other processes, including pollen and seed flow and genetic drift, reduce selection efficacy. The polymorphism (*π_a_*) at a nonsynonymous site is affected by the joint effects of selfing with gametophytic or sporophytic selection. Likewise, the ratio of nonsynonymous to synonymous polymorphism (*π_a_/π_s_*) is also affected by the same joint effects. Gene flow and genetic drift have opposite effects on *π_a_* or *π_a_/π_s_* in interacting with gametophytic and sporophytic selection. We discuss implications of this theory for detecting natural selection in terms of *K_a_/K_s_* and for interpreting the evolutionary divergence among gametophyte-specific, sporophyte-specific, and pleiotropic genes.

**Significance**

This study develops a new theory of how the ratio of nonsynonymous to synonymous divergence (*K_a_/K_s_*) changes under phase variation of selection during the plant life cycle. Effects of selfing on *K_a_/K_s_* are emphasized.

## Introduction

$K_{a}$ (or $K_{s}$) is the average number of nonsynonymous (or synonymous) nucleotide differences between protein-coding gene sequences per nonsynonymous (or synonymous) site. The relative rate of synonymous to nonsynonymous divergence per nucleotide site, $K_{a}/K_{s}$, is often used to detect natural selection occurring in a protein-coding gene at the evolutionary time scale. Neutrality or selection (positive or purifying) is signaled when $K_{a}/K_{s}$ is equal to or unequal to 1 (>1 or <1), respectively. Given a gene sequence, selection may exhibit “spatial” variation across nonsynonymous sites in both the type (positive and purifying) and strength ($K_{a}/K_{s}$ values) of selection along the sequence. Such spatial variation of selection could partly be cancelled out if the analysis is based on individual genes, such as the branch model from phylogenetic analysis with maximum likelihood (PAML) package in which each branch is hypothesized to have the same $K_{a}/K_{s}$ at any nonsynonymous site, and could yield purifying selection at the gene level (Yang 2006). Here, we demonstrate another type of variation of selection that occurs between gametophyte and sporophyte phases during the plant life cycle. This phase variation in terms of $K_{a}/K_{s}$ could influence detection of natural selection as well.

Phase variation in $K_{a}/K_{s}$ could arise from multiple processes. One is the potentially divergent selection between gametophyte and sporophyte phases during the life cycle. Haldane (1932) and Haldane and Jayakar (1963) thought that antagonistic selection could occur between two phases where one allele is favorable in the gametophyte phase but deleterious in the sporophyte phase, or vice versa. Theoretical studies show that antagonistic selection helps to maintain polymorphisms (Damgaard et al. 1994; Immler et al. 2012; Otto et al. 2015; Peters and Weis 2018). An alternative type is synergistic selection between gametophyte and sporophyte phases, which purges deleterious alleles but enhances positive selection of advantageous alleles (Walsh and Charlesworth 1992; Charlesworth and Charlesworth 1992; Damgaard 2000). Gametophytic selection can increase sporophyte fitness (Mulcahy and Mulcahy 1987; Winsor et al. 1987; Beaudry et al. 2020). Both types of selection can cause phase variation in $K_{a}/K_{s}$.

Evidence supports different types of selection in gametophytic and sporophytic phases. Phase-specific selection could likely occur in genes that are only expressed in the gametophyte or the sporophyte phase (Page and Grossniklaus 2002; Honys and Twell 2003; Rutley and Twell 2015; Liu et al. 2015; Zhang et al. 2021). Synergistic selection can be observed for genes that are co-expressed in both phases (Tanksley et al. 1981; Borg et al. 2009; Arunkumar et al. 2013; Frank and Scanlon 2015), whereas antagonistic selection is likely to occur in genes that are differentially expressed in both phases (Gossmann et al. 2016; Beaudry et al. 2020). These empirical observations provide a biological basis for modeling and testing phase variation of $K_{a}/K_{s}$.

The second process is the joint effects of selfing and biphasic selection, which could alter $K_{a}/K_{s}$ in a complicated way. Selfing enhances the purging of deleterious alleles via reducing the frequency of heterozygotes that mask deleterious alleles in the sporophyte phase. Selfing affects the mutation load in metapopulations (Roze and Rousset 2004) and, in the case of gametophytic selection, will have a strong effect on allele frequencies in gametes (Hu et al. 2019). Previous theory indicates that selfing/inbreeding favors the gametophyte generation, while outcrossing favors the sporophyte generation (Otto and Marks 1996). This can change the relative selection strengths between the two phases in various plant species and causes phase variation of $K_{a}/K_{s}$ throughout the life cycle.

The third process is the joint effects of gene flow and biphasic selection (Hu et al. 2019). Pollen flow directly influences gametophytic selection through its effects on the frequencies of deleterious alleles but indirectly affects sporophytic selection through the mating system. Seed flow directly interacts with sporophytic selection through introducing both homozygotes and heterozygotes to recipient populations. Both pollen and seed flow could bring about migration loads (Wright 1977; Hu and Li 2003; Lopez et al. 2008) and reduce selection efficacy. In addition, because $K_{a}/K_{s}$ is related to the fixation of alternative alleles in genetically distinct populations (Yang 2006; Kryazhimskiy and Plotkin 2008), it is of significance to examine how gene flow impedes the speciation process. Gene flow via haploid pollen and diploid seeds as vectors could also be responsible for the observed phase variation of $K_{a}/K_{s}$ during the life cycle.

Other processes, such as the interaction of mating system with genetic drift (Glemin 2007; Glemin and Muyle 2014) or with mutation, could alter $K_{a}/K_{s}$ (Li et al. 2023). Genetic drift enhances fixation of deleterious alleles and hence reduces selection efficacy (Kimura 1962), which makes $K_{a}/K_{s}$ for deleterious alleles approach 1.0. Selfing species are hypothesized to have a greater $K_{a}/K_{s}$ ratio than outcrossing species. However, this ratio often refers to that measured in the sporophyte phase rather than in the gametophyte phase. Thus, it is of significance to study how different models of biphasic selection change $K_{a}/K_{s}$ under multiple processes in both theory and practice. For comparison, we also consider $\pi_{a}/\pi_{s}$ where $\pi_{a}$ and $\pi_{s}$ are the average heterozygosities at the nonsynonymous and synonymous sites, respectively. Conceptually, these two ratios reflect different time scales, with the $K_{a}/K_{s}$ ratio measuring the long-term divergence of fixed alleles and the $\pi_{a}/\pi_{s}$ ratio measuring either short- or long-term polymorphisms of coexisting alleles. Empirical studies showed that these two ratios differ in characterizing molecular evolution and speciation (Wang et al. 2021; Li et al. 2023). It is interested to compare the similarity and differences between the two ratios under different models of selection.

Previous theories relevant to this topic include deterministic or stochastic selection in haploid and diploid phases in a random mating system, with emphasis on the conditions for maintaining polymorphisms (Scudo 1967; Ewing 1977), or fixation/loss of an allele or equilibrium (Hartl 1976). The theory also includes the haplodiploid selection model that has the same population genetic consequences as the X-linked genes in sexual selection model (Lester and Selander 1979; Avery 1984; Hall and Goodisman 2012), the haploid competition controlled by parents (Otto et al 2015), and the sex-specific selection where sexually antagonistic or synergistic selection takes place (Connallon and Calsbeek 2020; Lund-Hansen et al. 2021). Theory for maintaining biphasic cycles, including antagonistic selection, is also developed through analyzing the interplay between genetic and ecological effects (Rescan et al. 2016) or the unequal haploid and diploid fitness (Scott and Rescan 2016). Here, we develop new models of selection in gametophyte and sporophyte phases of plant life cycle, with emphasis on molecular evolution measured by $K_{a}/K_{s}$ and $\pi_{a}$ or $\pi_{a}/\pi_{s}$. We begin by describing the model of antagonistic selection and then the model of synergistic selection. A mainland–island model of population structure is employed to look at the effects of pollen and seed flow on these two measures. We finally discuss potential implications of the new theory.

## The Theory

### Methodology

**Fig. 1.**
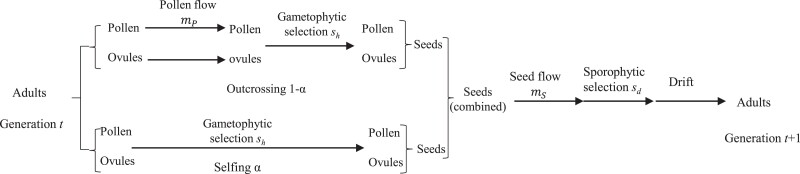
The life cycle used in model. The sequential events are indicated in plant life cycle, including generation of pollen and ovules, pollen flow, selection in the gametophyte phase, mixed mating, seed flow, selection in the sporophyte phase, and genetic drift. The arrow lines show occurrence of sequential events.

The model deals with a hermaphrodite plant species in a conventional mainland–island model. The mainland population is sufficiently large in size and stable in genetic composition. Only unidirectional gene flow from the mainland to the island is considered. Figure 1 shows the sequential events of life cycle modeled. We consider weak selection in both haploid gametophyte and diploid sporophyte phases and the same order of migration rate and drift effects. The terms with the second or higher order of these parameters are neglected for mathematic tractability. Effects of mutation on $K_{a}/K_{s}$ and $\pi_{a}/\pi_{s}$ at a nucleotide site are neglected in this study. The variables and parameters used in the model are summarized in supplementary table S1, Supplementary Material online.

Consider a biallelic site in autosomes, with an ancestral allele *A* and a mutant allele *a*. Two selection schemes are analyzed in the gametophyte and sporophyte phases (table 1). In the antagonistic selection scheme, allele *A* is deleterious in the gametophyte phase but favorable in the sporophyte phase, while allele *a* is favorable in the gametophyte phase but deleterious in the sporophyte phase. This selection scheme facilitates maintenance of polymorphisms (Haldane 1932; Haldane and Jayakar 1963), but genetic drift could yield fixation of the mutant allele. In the synergistic selection scheme, the mutant allele is deleterious in both gametophytic and sporophytic phases but can be fixed in a small population (Kimura 1962).

**Table 1:** Two Selection Schemes in Gametophyte and Sporophyte Phases

Selection Scheme	Gametophytic Selection		Sporophytic Selection		
	*A*	*a*	*AA*	*Aa*	*aa*
Antagonistic selection	$1-s_{h}$	1	1	$1-hs_{d}$	$1-s_{d}$
Synergistic selection	1	$1-s_{h}$	1	$1-hs_{d}$	$1-s_{d}$

Based on the life cycle modeled (fig. 1), we derive systematic change of allele frequency, which is caused by gene flow and biphasic selection. Following Wright's idea (Wright 1969), we derive the changes in allele frequencies due to selfing and outcrossing separately and then combine them to calculate genotypic frequencies before seed flow. The diffusion model is then applied to derive the fixation probability of the mutant allele and the density distribution of its frequency after genetic drift (Kimura 1962). We focus on $K_{a}/K_{s}$ and $\pi_{a}$ (or $\pi_{a}/\pi_{s}$ in the case with polymorphisms at a neutral site) in the island population under multiple scenarios. Based on the general analytical formulae, numerical examples are used to illustrate the patterns of $K_{a}/K_{s}$ and $\pi_{a}$ or $\pi_{a}/\pi_{s}$. Two Mathematica notebooks are provided for calculating $K_{a}/K_{s}$ and $\pi_{a}$ or $\pi_{a}/\pi_{s}$ for numerical analyses (Wolfram 1996).

### Antagonistic Selection

#### General Case

Let $p_{AA}$, $p_{Aa}$, and $p_{aa}$ be the genotypic frequencies in current adults in the island population. The per-generation change of gene frequency is derived below according to the life cycle (fig. 1). For the outcrossing part, with a probability of 1−α, the fitness of gametes *A* and *a* is set as $w_{A}=1-s_{h}$ and $w_{a}=1$ in both pollen and ovules, respectively, where $s_{h}$ is the selection coefficient. The average fitness in pollen is calculated as $w_{P}=w_{A}p_{A}^{*}+w_{a}p_{a}^{*}$, where $p_{A}^{*}$ and $p_{a}^{*}$ are the allele frequencies after pollen flow. The average fitness in ovules is $w_{O}=w_{A}p_{A}+w_{a}p_{a}$ where $p_{A}$ and $p_{a}$ are the allele frequencies in adults because ovules do not migrate. Genotypic frequencies in seeds are then calculated according to the random combination between pollen and ovules.

For the selfing part, with a probability of α, gametophytic selection only occurs in heterozygotes. The mean fitness in pollen or ovules is $1-\frac{1}{2}s_{h}$. Genotypic frequencies are calculated in seeds produced by selfing. Following Wright's (1969) idea, the overall genotypic frequencies in seeds are obtained by combining the selfing and outcrossing parts.

Genotypic frequencies after seed flow are then calculated. For antagonistic selection (table 1), the fitness of three genotypes is set as $W_{AA}=1$, $W_{Aa}=1-hs_{d}$, and $W_{aa}=1-s_{d}$ where $s_{d}$ is the selection coefficient and *h* is the degree of dominance. Allele *a* is completely masked by allele *A* in fitness when *h* = 0. Strong selection against allele *a* occurs when *h* = 1 (complete dominance). The additive selection model occurs in the sporophyte phase when *h* = 1/2. The average fitness in the sporophyte phase is $\bar{W}=W_{AA}p_{AA}^{**}+W_{Aa}p_{Aa}^{**}+W_{aa}p_{aa}^{**}$, where $p_{ij}^{**}$ is the frequency of genotype *ij* after seed flow. The per-generation systematic changes in gene and genotypic frequencies are derived in detail in supplementary appendix S1, Supplementary Material online in the Supplementary material.

For a mixed mating system, an equilibrium relationship for a single locus between the inbreeding coefficient *F* and the selfing rate α is approximated by $F=\frac{\alpha }{2-\alpha }$ (Haldane 1924; Wright 1969). According to equation (S1.22) of supplementary appendix S1, Supplementary Material online, the per-generation systematic change in allele frequency for the mutant allele is expressed as


(1) $$\begin{array}{rl} M_{\Delta p_{a}} & =p_{a}^{***}-p_{a}\\ & =\tilde{m}(Q_{a}-p_{a})+s_{h}p_{A}p_{a}\left(1-\frac{1}{2}\alpha (1+F)\right)\\ & \;-s_{d}p_{A}p_{a}\left(\;p_{a}+\frac{1}{2}\alpha p_{A}(1+F)+h(\;p_{A}-p_{a})\left(1-\frac{1}{2}\alpha (1+F)\right)\right), \end{array}$$

where $p_{a}^{***}$ is the allele frequency after sporophytic selection, $\tilde{m}$ stands for a composite migration rate, and $\tilde{m}=m_{S}+\frac{1-\alpha }{2}m_{P}$, in which $m_{P}$ and $m_{S}$ are the migration rates of pollen and seeds from the mainland population, respectively. $Q_{a}$ ($Q_{A}+Q_{a}=1$) is the frequency of allele *a* in migrants from the mainland population. The per-generation systematic change in equation (1) is caused by gene flow, gametophytic selection, and sporophytic selection. The per-generation systematic change for allele frequency $p_{A}$, $M_{\Delta p_{A}}$, is equal to the negative $M_{\Delta p_{a}}$, that is, $M_{\Delta p_{A}}=-M_{\Delta p_{a}}$.

We now incorporate the genetic drift effects into the change of allele frequencies (fig. 1). Let $N_{e}$ be the effective size of the island population. The genetic drift process generates variation of allele frequency but does not change the mean allele frequency. The variance for the per-generation change of gene frequency $\Delta p_{a}$ is given by


(2) $$\begin{array}{rl} V_{\Delta p_{a}} & =\frac{\;p_{a}^{***}(1-p_{a}^{***})}{2N_{e}}\\ & \approx \frac{\;p_{a}(1-p_{a})}{2N_{e}}. \end{array}$$

Similarly, the variance for the per-generation change of allele frequency, $\Delta p_{A}$, is equal to $V_{\Delta p_{a}}$ according to the binomial distribution of allele frequency, $V_{\Delta p_{A}}=V_{\Delta p_{a}}$.

From Caballero and Hill (1992), the effective population size in a mixed mating system is re-expressed as $N_{e}=\frac{N}{1+F}=\left(1-\frac{1}{2}\alpha \right)N$ under our assumption of a single locus without background selection, where *N* is the actual population size. Selfing reduces the effective population size by a maximum rate of 50%.

According to Kimura (1962), the fixation probability of the mutant allele, $\varphi (\;p_{0})$, with an initial frequency $p_{0}$ in a population of effective size $N_{e}$ is calculated as


(3) $$\varphi (\;p_{0})=\frac{\int_{0}^{\;p_{0}}\;G(\;p_{a})dp_{a}}{\int_{0}^{1}\;G(\;p_{a})dp_{a}},$$

where


(4) $$G(\;p_{a})=exp\left(-2\int \frac{M_{\Delta p_{a}}}{V_{\Delta p_{a}}}dp_{a}\right)\;.$$

Bessho and Otto (2017) provide an alternative formula for calculating the fixation probability for a selected allele with haploid–diploid life cycles under random mating only. In their case, the fixation probability is calculated by weighting the fixation probabilities in haploid and diploid phases with their fractions, which is different from the preceding calculation.

Substitution of equation (1) and equation (2) into equation (4) yields


(5) $$\begin{array}{rl} G(\;p_{a})\;= & (1-p_{a}{)}^{-2(2-\alpha )N\tilde{m}Q_{A}}p_{a}^{-2(2-\alpha )N\tilde{m}Q_{a}}\cdot \\ & \exp(2(1-\alpha )(1-2h)Ns_{d}p_{a}^{2}+2N((\alpha +2(1-\alpha )h)s_{d}\\ & -2(1-\alpha )s_{h})p_{a}). \end{array}$$

For antagonistic selection, the mutant allele (*a*) could attain a high frequency or approach fixation, depending upon the relative effects of selection, migration, and genetic drift.

If the mutant allele is neutral $\left(s_{h}=s_{d}=0\right)$, that is, in the case of a synonymous site, its fixation probability with an initial frequency of $p_{0}$ is


(6) $$\varphi_{0}(\;p_{0})=\frac{\int_{0}^{\;p_{0}}\;p_{a}^{-2(2-\alpha )N\tilde{m}Q_{a}}{\left(1-p_{a}\right)}^{-2(2-\alpha )N\tilde{m}Q_{A}}dp_{a}}{\int_{0}^{1}\;p_{a}^{-2(2-\alpha )N\tilde{m}Q_{a}}{\left(1-p_{a}\right)}^{-2(2-\alpha )N\tilde{m}Q_{A}}dp_{a}}.$$

When the island population is completely isolated from the mainland population ($\tilde{m}=0$), gene frequency in the island population is operated by the genetic drift only. The fixation probability is $\varphi_{0}(\;p_{a})=\frac{\int_{0}^{\;p_{0}}\;1dp_{a}}{\int_{0}^{1}\;1\;dp_{a}}=p_{0}$, which equals the initial allele frequency, for example, $\varphi_{0}\left(\frac{1}{2N}\right)=\frac{1}{2N}$ (Kimura 1962).

Let *u* be the mutation rate from allele *A* to *a* at the nonsynonymous site, without backward mutation. The substitution rate with allele *a* at the nonsynonymous site, measured by $K_{a}$, is calculated as


(7) $$K_{a}=2N_{e}u\;*\;\varphi (\;p_{0}),$$

where $2N_{e}u$ is the expected number of nonsynonymous mutations (mutant allele *a*) entering the population per generation and $\varphi (\;p_{0})$ is the fixation probability of the mutant allele with initial frequency $p_{0}$ (Bustamante 2005; Kryazhimskiy and Plotkin 2008). $K_{a}$ measures the rate of nonsynonymous substitution between gene sequences per nonsynonymous site.

Let the substitution rate per synonymous site (the neutral allele), $K_{s}$, be calculated as


(8) $$K_{s}=2N_{e}\mu \;*\;\varphi_{0}(\;p_{0}).$$

The $2N_{e}\mu$ is the expected number of synonymous (neutral) mutations entering the population per generation, and $\varphi_{0}(\;p_{0})$ is the fixation probability of a neutral mutant with initial frequency $p_{0}$. For a completely isolated population ($\tilde{m}=0$), $K_{s}$ is equal to the mutation rate, $K_{s}=2N_{e}\mu \times \frac{1}{2N_{e}}=\mu$. If both migration and drift processes are involved, $K_{s}$ is calculated by substituting equation (6) into equation (8).

Denote a ratio of the evolutionary rate at the nonsynonymous site relative to that at the neutral site by $\frac{K_{a}}{K_{s}}$, which is calculated by


(9) $$\frac{K_{a}}{K_{s}}=\frac{\varphi (\;p_{0})}{\varphi_{0}(\;p_{0})}\cdot \frac{u}{\mu }.$$

Unequal mutation rates (u≠μ) also influence $K_{a}/K_{s}$, which is not focused on here.

We now consider polymorphisms at nonsynonymous and synonymous sites ($\pi_{a}$ or $\pi_{a}/\pi_{s}$). Under the joint effects of migration, selection, and genetic drift, the density distribution of allele frequency at equilibrium is calculated as $\phi (\;p_{a})=\frac{C}{V_{\Delta p_{a}}}\exp\;\left(2\int \frac{M_{\Delta p_{a}}}{V_{\Delta p_{a}}}dp_{a}\right)=\frac{C}{V_{\Delta p_{a}}G(\;p_{a})}$ (Kimura 1962), where *C* is the constant that satisfies $\overset{1}{\underset{0}{\int }}\;\phi (\;p_{a})dp_{a}=1$ (Wright 1969). Substitution of equations (1) and (2) into $\phi (\;p_{a})$ yields the following expression:


(10) $$\begin{array}{rl} \phi (\;p_{a})\;= & C(2-\alpha )N\cdot \\ & exp(-2N(1-\alpha )(1-2h)s_{d}p_{a}^{2}-2N((\alpha +2h(1-\alpha ))s_{d}\\ & -2(1-\alpha )s_{h})p_{a})\cdot p_{a}^{2N(2-\alpha )\tilde{m}Q_{a}-1}(1-p_{a}{)}^{2N(2-\alpha )\tilde{m}Q_{a}-1}. \end{array}$$

The expected allele frequency, ${\bar{\;p}}_{a}$, is numerically calculated by ${\bar{\;p}}_{a}=\overset{1}{\underset{0}{\int }}\;p_{a}\phi (\;p_{a})dp_{a}$, and ${\bar{\;p}}_{A}$ is equal to 1 − ${\bar{\;p}}_{a}$.

Under gametophytic and/or sporophytic selection, when both alleles may coexist in the island population, the average heterozygosity per nonsynonymous site, $\pi_{a}$, is calculated as


(11) $$\begin{array}{rl} \pi_{a} & =\overset{1}{\underset{0}{\int }}\;2p_{a}(1-p_{a})\phi (\;p_{a})dp_{a}=2({\bar{\;p}}_{A}{\bar{\;p}}_{a}-V_{p}), \end{array}$$

where $V_{p}$ is the variance of allele frequency at equilibrium, which is induced by the genetic drift process.

At the neutral site ($s_{d}=s_{h}=0$), the density distribution of allele frequency of equation (10) is simplified as


(12) $$\begin{array}{rl} \phi_{0}(\;p_{a})=\frac{\Gamma (2N(2-\alpha )\tilde{m})}{\Gamma (2N(2-\alpha )\tilde{m}Q_{A})\Gamma (2N(2-\alpha )\tilde{m}Q_{a})}\\ & \;p_{a}^{2N(2-\alpha )\tilde{m}Q_{a}-1}(1-p_{a}{)}^{2N(2-\alpha )\tilde{m}Q_{A}-1} \end{array}.$$

The genetic diversity at the synonymous site is maintained by migration and genetic drift. The average heterozygosity per synonymous site, $\pi_{s}$, is calculated by


(13) $$\begin{array}{rl} \pi_{s} & =\overset{1}{\underset{0}{\int }}\;2p_{a}(1-p_{a})\phi_{0}(\;p_{a})dp_{a}\\ & =2Q_{A}Q_{a}\;\cdot \left(1-\frac{1}{1+2(2-\alpha )N\tilde{m}}\right), \end{array}$$

where the proportion in parentheses is the reduction due to genetic drift effects. When only genetic drift process operates on the neutral site, polymorphisms could be transiently maintained before any allele is lost or fixed (e.g., $\pi_{s}=0$).

In the following parts of this section, we consider specific cases, which can be applied to interpret the evolutionary divergence among genes expressed in gametophyte phase, sporophyte phase, and two phases. Additive and nonadditive selection models in the sporophyte phase are separately addressed.

#### Additive Selection (*h* = 1/2)

Consider a specific case where the island population is completely isolated from the mainland population ($\tilde{m}=0$). Let mutation rates be equal at synonymous and nonsynonymous sites (u=μ). Following Kimura (1962), we assume that the nonsynonymous site is evolved by selection and genetic drift processes. When only the gametophytic selection takes place ($s_{d}=0,\;\;s_{h}\neq 0$), the mutant allele (*a*) is favorable and is expected to be fixed although drift could slightly reduce the fixation probability (Kimura 1962). This case could occur for the genes that are expressed only in the gametophyte phase. The relative evolutionary rate is approximated by


(14) $$\frac{K_{a}}{K_{s}}\;=\frac{1-\exp(-4N(1-\alpha )s_{h}p_{0})}{1-\exp(-4N(1-\alpha )s_{h})}\cdot \frac{1}{\;p_{0}}.$$

The initial frequency of allele *a* at both the neutral and selective sites is set as $p_{0}$ in deriving (14). It can be shown that $K_{a}/K_{s}\;$ increases as $s_{h}$ increases. Let $\epsilon =4N(1-\alpha )s_{h}$. The partial differential of $\frac{K_{a}}{K_{s}}$ with respect to $s_{h}$ is


(15) $$\frac{\partial \left(\frac{K_{a}}{K_{s}}\right)}{\partial s_{h}}=\frac{4N(1-\alpha )}{\;p_{0}{\left(1-e^{-\epsilon }\right)}^{2}}e^{-\epsilon (1+p_{0})}(\;p_{0}e^{\epsilon }-p_{0}-e^{\epsilon p_{0}}+1),$$

which is greater than 0 from the results in supplementary appendix S2, Supplementary Material online. Similarly, a large population enhances gametophytic selection $\left(\partial \left(\frac{K_{a}}{K_{s}}\right)/\partial N>0\right)$ and facilitates the fixation of allele *a*. However, selfing reduces the selection efficacy $\left(\partial \left(\frac{K_{a}}{K_{s}}\right)/\partial \alpha <0\right)$ and hence reduces the substitution rate with allele *a*.

**Fig. 2.**
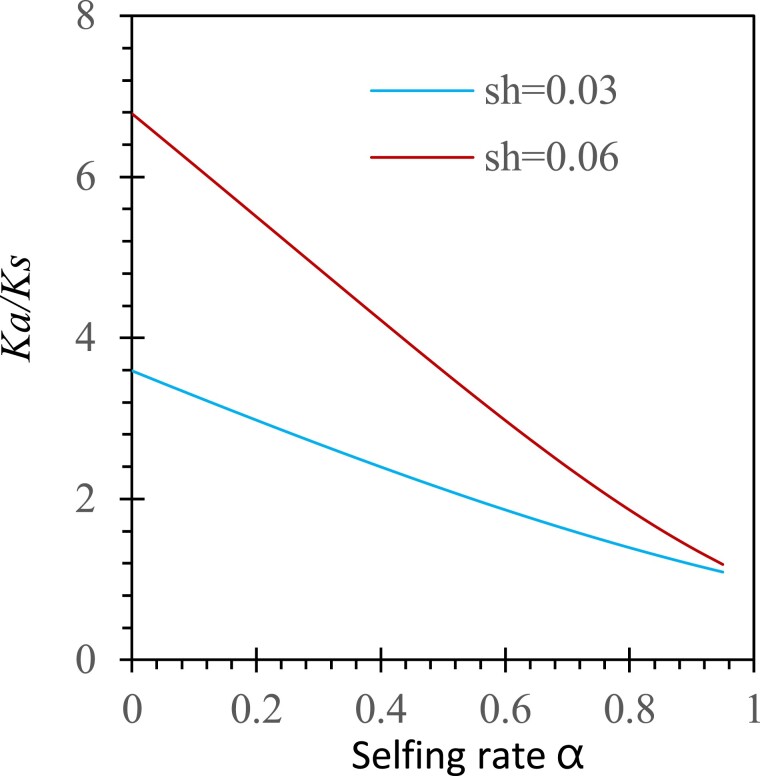
Examples of gametophytic selection changing $K_{a}/K_{s}$ under different selfing rates. Results are derived from equation (14) with the selection coefficients $s_{h}=0.03$ or 0.06 and $s_{d}=0$, the degree of dominance *h* = 0.5, the population size N=30, the initial frequencies $p_{0}=1/2N$, and the migration rates of seeds and pollen $m_{S}=m_{P}=0$.

Figure 2 shows two numerical examples under gametophytic selection only (*N* = 30, $s_{d}$ = 0, and $s_{h}$ = 0.03 or 0.06), with the initial frequency of the mutant allele $p_{0}=\frac{1}{2N}$. Selfing reduces the efficacy of gametophytic selection. Because the mutant allele approaches fixation despite the impeding effects from selfing, the level of polymorphism at this site decreases to 0 ($\pi_{a}=0$). Under the sole genetic drift process, the neutral site is polymorphic for finite time before the mutant allele is fixed or lost ($\pi_{s}=0$), and selfing accelerates this process because it reduces the effective population size.

When only sporophytic selection takes place ($s_{d}\neq 0,s_{h}=0$), the mutant allele *a* is deleterious and is under purifying selection. In this situation, the relative evolutionary rate is approximated by


(16) $$\frac{K_{a}}{K_{s}}=\frac{\exp\;(2Ns_{d}p_{0})-1}{\exp\;(2Ns_{d})-1}\cdot \frac{1}{\;p_{0}}.$$

The initial frequency of allele *a* at the neutral site is set as $p_{0}$ in deriving equation (16). A smaller population size and/or weaker selection pressure can impede the efficacy of purifying selection against the mutant allele, $\partial \left(\frac{K_{a})}{K_{s}}\right)/\partial N<0$ and $\partial \left(\frac{K_{a}}{K_{s}}\right)/\partial s_{d}<0$. If the initial allele frequency is $\;p_{0}=\frac{1}{2N}$, equation (16) reduces to the conventional result obtained by Kimura (1962). Note that *N* is the actual population size; equation (16) can also be re-expressed in terms of the effective population size through the relationship of $N=2N_{e}/(2-\alpha )$, that is


(17) $$\frac{K_{a}}{K_{s}}=\frac{4N_{e}s_{d}}{(2-\alpha )\left(\exp\left(\frac{4N_{e}s_{d}}{2-\alpha }\right)-1\right)}.$$

Selfing increases the evolutionary rate $K_{a}/K_{s}$$\left(\partial \left(\frac{K_{a}}{K_{s}}\right)/\partial \alpha >0\right)$ despite purifying selection against the mutant allele ($K_{a}/K_{s}<1$). This is realized through reducing effective population size rather than reducing the efficacy of gametophytic selection.

To compare the relative evolutionary rates between gametophyte- and sporophyte-specific genes, we assume comparable selection strength in two phases ($s_{d}=2s_{h}$). In the outcrossing system (α=0), the two phase-specific genes have equal absolute $K_{a}/K_{s}$ values from equations (14) and (16), given the same initial allele frequency of the mutant allele. However, in the mixed or selfing system (0<α≤1), which leads to $s_{d}=2s_{h}>2(1-\alpha )s_{h}$, the absolute $K_{a}/K_{s}$ in equation (14) is smaller than the absolute $K_{a}/K_{s}$ in equation (16), given their same initial allele frequencies. Gametophyte-specific genes are expected to have a lower absolute evolutionary rate than sporophyte-specific genes in the mixed mating system.

When both gametophytic selection and sporophytic selection take place, this likely occurs for those genes that are co-expressed in two phases. The evolutionary rate for the mutant allele is calculated by


(18) $$\frac{K_{a}}{K_{s}}=\frac{1-exp(2N(s_{d}-2(1-\alpha )s_{h})p_{0})}{1-exp(2N(s_{d}-2(1-\alpha )s_{h}))}\;\cdot \frac{1}{\;p_{0}}.$$

An increase in sporophytic selection coefficient ($s_{d}$) reduces $K_{a}/K_{s}$, whereas an increase in gametophytic selection coefficient ($s_{h}$) increases $K_{a}/K_{s}$. Equation (18) indicates that biphasic selection is completely offset in outcrossing (α=0) under comparable strengths of allelic selection ($s_{d}=2s_{h}$), which yields $K_{a}/K_{s}=1$ (note $G(p_{a})$ = 1 under these conditions). Selfing (α≠0) modifies the extent of offset by reducing the efficacy of gametophytic selection.

From equation (18), we obtain the following relationship:


(19) $$\frac{\partial \left(\frac{K_{a}}{K_{s}}\right)}{\partial s_{h}}=-2(1-\alpha )\frac{\partial \left(\frac{K_{a}}{K_{s}}\right)}{\partial s_{d}}.$$

Under comparable allelic selection between the two phases ($s_{d}=2s_{h}$), the responses of the evolutionary rate $K_{a}/K_{s}$ to the change in gametophytic and sporophytic selection are the same in the outcrossing system (α=0), that is, $\partial \left(\frac{K_{a}}{K_{s}}\right)/\partial s_{h}=\left|\partial \left(\frac{K_{a}}{K_{s}}\right)/\partial \left(\frac{s_{d}}{2}\right)\right|$. However, the mixed mating system (0<α<1) can lead to the gametophytic selection to be less efficient than the sporophytic selection, that is, $\partial \left(\frac{K_{a}}{K_{s}}\right)/\partial s_{h}<\left|\partial \left(\frac{K_{a}}{K_{s}}\right)/\partial \left(\frac{s_{d}}{2}\right)\right|$. Complete selfing (α=1) removes the gametophytic selection but enhances purifying selection against the mutant allele in the sporophyte phase.

**Fig. 3.**
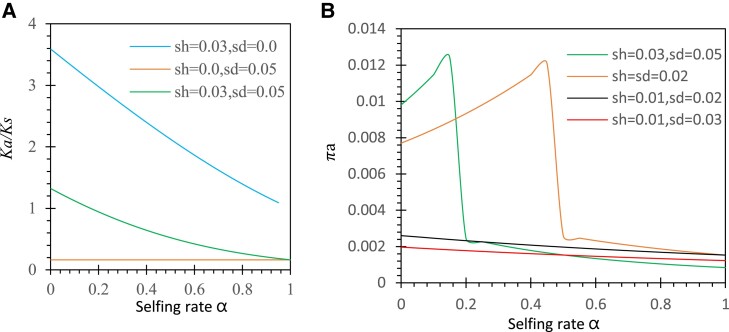
Examples of the patterns of $K_{a}/K_{s}$ and $\pi_{a}$ under antagonistic selection. Results are derived from a Mathematica notebook. In (*A*), three sets of selection parameter settings are the gametophytic selection ($s_{d}=0$ and $s_{h}=0.03$), the sporophyte selection ($s_{d}=0.05$ and $s_{h}=0.0$), and biphasic selection ($s_{d}=0.05$ and $s_{h}=0.03$). In (*B*), four biphasic selection settings are examined: $s_{d}=0.05$ and $s_{h}=0.03$; $s_{d}=s_{h}=0.02$; $s_{d}=0.02$, $s_{h}=0.01$; and $s_{d}=0.03$ and $s_{h}=0.01$. Other parameters are the population size N=30, the degree of dominance h=0.5, and the migration rates of seeds and pollen $m_{S}=m_{P}=0$.

Figure 3 *A* shows the pattern of $K_{a}/K_{s}$ for the mutant allele under the antagonistic selection compared with those under gametophytic and sporophyte selection alone. The results indicate that the opposite effects from antagonistic selection can partially offset with the given parameter settings ($s_{d}=0.05$ and $s_{h}=0.03$). Purifying selection in the sporophyte phase dominates and produces small $K_{a}/K_{s}$ under the condition of $s_{d}>2(1-\alpha )s_{h}$. Positive selection in the gametophytic phase dominates and produces large $K_{a}/K_{s}$ under the condition of $s_{d}<2(1-\alpha )s_{h}$. A complete offset is expected under the condition of $s_{d}=2(1-\alpha )s_{h}$, which leads to $K_{a}/K_{s}$ = 1 (neutral case).

Figure 3 *B* shows the pattern of $\pi_{a}$ as the selfing rate increases from 0 to 1. The $\pi_{a}$ value increases with the selfing rate under the condition of $s_{d}-2(1-\alpha )s_{h}<0$ but decreases with the selfing rate under the condition of $s_{d}-2(1-\alpha )s_{h}>0$. The turning point is located at the selfing rate $\alpha =1-s_{d}/2s_{h}$, where completely balancing selection occurs.

#### Nonadditive Selection (h≠1/2)

**Fig. 4.**
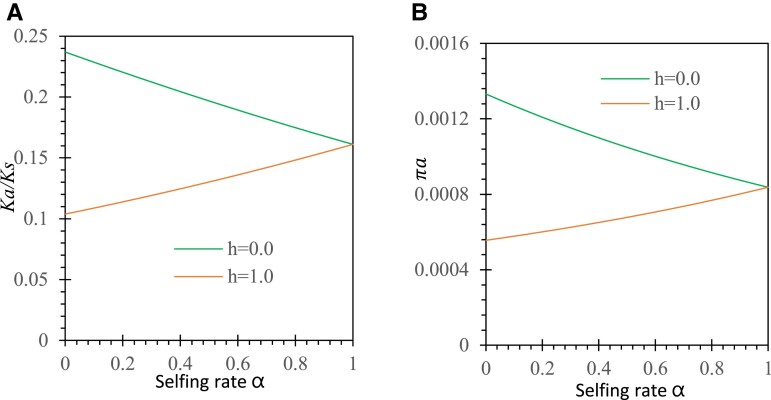
Examples of dominance effects on $K_{a}/K_{s}$ and $\pi_{a}$ under antagonistic selection. Results are derived from equations (3), (5), (8), (9), and (11): (*A*) $K_{a}/K_{s}$ ratio under *h* = 1 and 0 and (*B*) $\pi_{a}$ under *h* = 1 and *h* = 0. Other parameters are the selection coefficients $s_{d}=0.05$ and $s_{h}=0$, the population size N=30, the initial allele frequencies $p_{0}=1/2N$, and the migration rates of seeds and pollen $m_{S}=m_{P}=0$.

Under nonadditive selection in the sporophyte phase, we consider two specific cases (*h* = 0 or 1) to illustrate the effects of dominance selection on evolutionary rate. Figure 4*A* shows a comparison of the results under *h* = 1 versus *h* = 0 with the same initial allele frequency $p_{0}=1/2N$ and without gametophytic selection ($s_{h}$ = 0). Purifying selection against allele *a* is enhanced under complete dominance (*h* = 1) but weakened when allele *a* is completely masked by allele *A* in heterozygotes (*h* = 0). This results in a smaller $K_{a}/K_{s}$ under *h* = 1 than under *h* = 0. However, $K_{a}/K_{s}$ values tend to be the same in two cases as the selfing rate increases from 0 to 1. The $\pi_{a}$ value exhibits the pattern similar to that of $K_{a}/K_{s}$ as the selfing rate increases from 0 to 1 (fig. 4*B*). Complete dominance of the mutant allele over the ancestral allele (*h* = 1) has larger effects of purifying selection on the mutant allele in the selfing and mixed mating systems.

### Synergistic Selection

In the synergistic selection scheme, allele *a* is deleterious in both gametophyte and sporophyte phases but could be maintained under migration or has a small probability of fixation under large genetic drift effects (Kimura 1962). The fitness of gametes and zygotes is set in table 1. Under the mainland–island model, the per-generation systematic change of gene frequency, $M_{\Delta p_{a}}$, can be directly obtained by replacing positive $s_{h}$ in equation (1) with negative $s_{h}$. Although biphasic selection is in the same direction, selfing can regulate the impacts of gametophytic selection against the mutant allele (Hu and Li 2003; Hu 2015).

The variance for the per-generation change of allele frequency due to genetic drift, $V_{\Delta p_{a}}$, remains the same as equation (2). The fixation probability of allele *a*, $\varphi (\;p_{0})$, is calculated using equation (3) except that $G(\;p_{a})$ is calculated using a negative $s_{h}$ in equation (5).

$K_{a}/K_{s}$ for the mutant allele is numerically calculated using equation (9) under equal mutation rates between synonymous and nonsynonymous sites. The distribution of allele frequency at equilibrium, $\phi (\;p_{a})$, is calculated by replacing the negative $s_{h}$ in equation (10) with positive $s_{h}$. The average heterozygosity at the nonsynonymous site, $\pi_{a}$, is calculated using equation (11). The average heterozygosity at the synonymous site, $\pi_{s}$, is given by equation (13) when polymorphisms are maintained by migration and drift processes. Using these equations, we numerically evaluate $\pi_{a}$ or $\pi_{a}/\pi_{s}$ in different cases.

#### Additive Selection (*h* = 1/2)

Consider a specific case where the island population is completely isolated from the mainland population. Under the additive selection model (h=1/2) and equal mutation rates at synonymous and nonsynonymous sites (u=μ), $K_{a}/K_{s}$ is derived as


(20) $$\frac{K_{a}}{K_{s}}=\frac{1-exp(2N(s_{d}+2(1-\alpha ))s_{h})p_{0})}{1-exp(2N(s_{d}+2(1-\alpha ))s_{h}))}\frac{1}{\;p_{0}}.$$

Unlike the case of antagonistic selection, equation (20) indicates the cumulative effects of biphasic selection on $K_{a}/K_{s}$. When the initial frequency is $p_{0}=\frac{1}{2N}$, equation (20) is approximated by $\frac{K_{a}}{K_{s}}=\frac{2N(s_{d}+2(1-\alpha )s_{h})}{e^{2N(s_{d}+2(1-\alpha )s_{h})}-1}$, indicating that selfing reduces the contribution of gametophytic selection to $K_{a}/K_{s}$ because of the negative selfing (α) effects in the term of gametophytic selection.

Similarly, under comparable selection strength in two phases ($s_{d}=2s_{h}$), the gametophyte- and sporophyte-specific genes have equal $K_{a}/K_{s}$ values in the outcrossing system (α=0), given the same initial allele frequency of the mutant allele. However, in the mixed or selfing system (0<α≤1), which leads to $s_{d}=2s_{h}>2(1-\alpha )s_{h}$, $K_{a}/K_{s}$ for gametophyte-specific genes is smaller than that for sporophyte-specific genes, given their same initial allele frequencies.

For genes undergoing gametophytic selection only, $K_{a}/K_{s}$ increases as the selfing rate increases (supplementary fig. S1*A*, Supplementary Material online). Selfing impedes gametophytic selection against the mutant allele. For genes undergoing sporophytic selection only, $K_{a}/K_{s}$ for the mutant allele has the same expression as equation (15) or (16) in antagonistic selection and increases as the selfing rate increases in terms of effective population size. However, $K_{a}/K_{s}$ is not sensitive to the selfing rate in terms of actual population size (supplementary fig. S1*A*, Supplementary Material online). For genes undergoing biphasic selection, cumulative effects reinforce purifying selection against the mutant allele (supplementary fig. S1*A*, Supplementary Material online).

The polymorphism at the nonsynonymous site, $\pi_{a}$, exhibits the patterns similar to those of $K_{a}/K_{s}$ as the selfing rate increases from 0 (outcrossing) to 1 (selfing) (supplementary fig. S1*B*, Supplementary Material online). Biphasic selection further reduces polymorphic level, compared with those under single-phase selection.

For genes undergoing biphasic selection, comparable strengths of selection ($s_{d}=2s_{h}$) lead to equal contribution from each phase to the evolutionary rate in outcrossing systems (α=0). However, selfing reduces the proportion of gametophytic selection in a mixed mating system (α≠0). Similarly, we obtain the following relationship:


(21) $$\partial \left(\frac{K_{a})}{K_{s}}\right)/\partial s_{h}=(1-\alpha )\partial \left(\frac{K_{a}}{K_{s}}\right)/\partial \left(\frac{s_{d}}{2}\right).$$

Selfing reduces sensitivity of $K_{a}/K_{s}$ to the change of gametophytic selection, resulting in a lower efficiency of gametophytic selection than sporophytic selection.

From equation (20), the response of $K_{a}/K_{s}$ to the change of the selfing rate is


(22) $$\partial \left(\frac{K_{a}}{K_{s}}\right)/\partial \alpha =\frac{4Ns_{h}e^{x(1+p_{0})}}{{\left(e^{-x}-1\right)}^{2}p_{0}}(\;p_{0}e^{-x}-p_{0}-e^{-xp_{0}}+1),$$

where $x=2N(s_{d}+2(1-\alpha )s_{h})$. From supplementary appendix S2, Supplementary Material online, this response is positive (>0), indicating that selfing increases $K_{a}/K_{s}$ through reducing the efficacy of gametophytic selection.

#### Nonadditive Selection (h≠1/2)

**Fig. 5.**
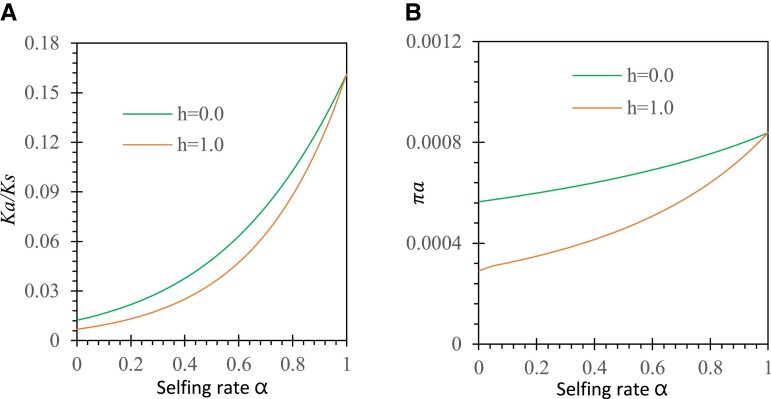
Examples of dominance effects on $K_{a}/K_{s}$ and $\pi_{a}$ under synergistic selection. Results are derived from a Mathematica notebook. The parameters except for the degree of dominance (*h* = 0 and 1) are the gametophytic selection ($s_{h}=0.03$), sporophytic selection ($s_{d}=0.05$), the population size N=30, the initial allele frequencies $p_{0}=1/2N$, and the migration rates of seeds and pollen $m_{S}=m_{P}=0$.

For genes undergoing sporophyte selection only ($s_{h}$ = 0, $s_{d}\neq 0$), the same patterns as those in the case of antagonistic selection ($s_{h}$ = 0, $s_{d}\neq 0$) are expected for the change of $K_{a}/K_{s}$ or $\pi_{a}$ with the selfing rate. This is because $G(\;p_{a})$ and $\phi (\;p_{a})$ functions are the same between the two cases. Figure 5*A* shows that selfing enhances $K_{a}/K_{s}$ under biphasic selection. As expected, the complete underdominance of the ancestral allele over the mutant allele (*h* = 1) gives great selection pressure against the mutant allele. When the ancestral allele completely masks the mutant allele in heterozygotes (*h* = 0), selection pressure is alleviated, which produces $K_{a}/K_{s}$ slightly higher than that under *h* = 1.

Figure 5 *B* shows that selfing impedes gametophytic selection but increases $\pi_{a}\;$ under biphasic selection. Polymorphism is enhanced when the mutant allele is completely masked by the ancestral allele (*h* = 0). As expected, when the mutant allele is underdominant (*h* = 1), polymorphism is reduced.

Compared with the results from sporophyte selection only (fig. 4), the presence of gametophytic selection adds additional selection against the mutant allele (fig. 5). Both $K_{a}/K_{s}$ and $\pi_{a}$ decrease in a mixed mating system (0<α<1).

### Pollen and Seed Flow

**Fig. 6.**
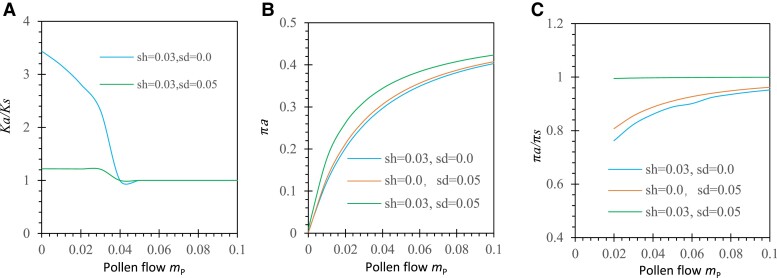
Examples of the effects of pollen flow on $K_{a}/K_{s}$ and $\pi_{a}$ or $\pi_{a}/\pi_{s}$ under antagonistic selection in a predominantly outcrossing system (*α* = 5%). Results are derived from a Mathematica notebook: (*A*) $K_{a}/K_{s}$; (*B*) $\pi_{a}$; and (*C*) $\pi_{a}/\pi_{s}$. The parameters except for selection coefficients shown in figures are the population size *N* = 30, the initial allele frequencies $p_{0}=1/2N$, the degree of dominance *h* = 0.5, the migration rate of seeds $m_{S}=0$, and the migrant allele frequencies $Q_{A}=Q_{a}=0.5$.

Gene flow with higher polymorphisms in migrants counteracts directional selection in the recipient population (Wright 1969). This impedes fixation of mutant or ancestral alleles but may increase polymorphisms ($\pi_{a}$ or $\pi_{a}/\pi_{s}$). To examine effects of pollen flow, we consider a predominantly outcrossing species that allows pollination from alien pollen. For instance, let the migrant allele frequency $Q_{a}$ = 0.5 in the mainland population, with the maximum polymorphism. Under antagonistic selection, fixation of either the mutant allele or the ancestral allele is very small under constant rate of pollen or seed flow. Figure 6*A* shows that, when the mutant allele is under gametophytic ($s_{h}$ = 0.03, $s_{d}$ = 0, and α = 5%) or biphasic selection ($s_{h}$ = 0.03, $s_{d}$ = 0.05, and α = 5%), the evolution rate ($K_{a}/K_{s}$) decreases to 1 as the migration rate of pollen increases from 0 to 0.1. When the mutant allele is deleterious ($s_{h}$ = 0, $s_{d}$ = 0.05, and α = 5%), $K_{a}/K_{s}$ (<1) also decreases as the migration rate of pollen increases (note that $G(\;p_{a})$ is not integrable using Mathematica notebooks when $m_{P}$ is greater than 0.04 with the parameter settings). Figure 6*B* shows that $\pi_{a}$ increases as the migration rate of pollen increases from 0 to 0.1. Likewise, $\pi_{a}/\pi_{s}$ also increases as the migration rate of pollen increases (fig. 6*C*). Biphasic selection enhances $\pi_{a}/\pi_{s}$ compared with the case of single-phase selection. Although seed flow is not restricted by the type of mating system, it generates the patterns similar to those generated by pollen flow in changing $K_{a}/K_{s}$ and $\pi_{a}$ or $\pi_{a}/\pi_{s}$ (supplementary fig. S2, Supplementary Material online).

Under synergistic selection, gene flow reduces $K_{a}/K_{s}$ but increases $\pi_{a}$ or $\pi_{a}/\pi_{s}$. In the predominantly outcrossing system (e.g., α = 5%), $K_{a}/K_{s}$ decreases as $m_{P}$ increases from 0 to 0.03 under both single-phase selection and biphasic selection (supplementary fig. S3, Supplementary Material online). Polymorphisms in terms of $\pi_{a}$ and $\pi_{a}/\pi_{s}$ increase as the migration rate of pollen increases from 0 to 0.1 (supplementary fig. S3, Supplementary Material online). Unlike the case of antagonistic selection, cumulative effects are present under biphasic selection (supplementary fig. S3, Supplementary Material online). Similarly, seed flow generates the patterns similar to those generated by pollen flow in changing $K_{a}/K_{s}$, $\pi_{a}$, and $\pi_{a}/\pi_{s}$ (supplementary fig. S4, Supplementary Material online). The difference between seed and pollen flow is due to larger effects of seed flow, given their same migration rates.

### Genetic Drift

**Fig. 7.**
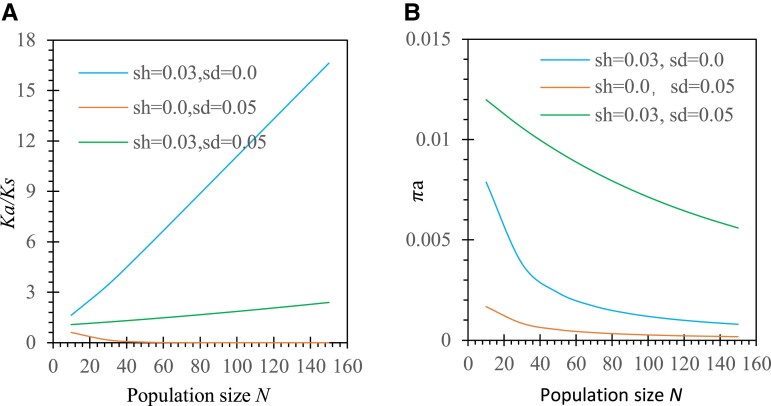
Examples of genetic drift effects $K_{a}/K_{s}$ and $\pi_{a}$ under antagonistic selection in a predominantly outcrossing system (*α* = 5%): (*A*) $K_{a}/K_{s}$ and (*B*) $\pi_{a}$. Results are derived from a Mathematica notebook. Parameters except for the selection coefficient and population size shown in figures are the initial allele frequencies $p_{0}=1/2N,\;$ the degree of dominance *h* = 0.5, and the migration rates of pollen and seeds $m_{P}=m_{S}=0$.

To evaluate the effects of genetic drift, we fix all parameters except for changing population size (*N*) in the isolated island population ($m_{P}=m_{S}=0$). As expected, a large population improves selection efficiency but still generates different patterns of evolutionary rates between the two selection schemes. Under antagonistic selection, $K_{a}/K_{s}$ substantially increases as the population size increases for genes undergoing gametophytic selection only (fig. 7*A*). This is because positive selection for the mutant allele is enhanced in larger populations. For genes undergoing sporophytic selection only, larger populations slightly reduce $K_{a}/K_{s}$ because the power of purging deleterious alleles increases (fig. 7*A*). For genes undergoing biphasic selection and additive selection model (*h* = 1/2) in the sporophyte phase, $K_{a}/K_{s}$ could increase as the population increases under the condition of $s_{d}-2(1-\alpha )s_{h}<0$ (fig. 7*A*) or decrease under the condition of $s_{d}-2(1-\alpha )s_{h}>0$ (data not provided here). Opposite effects between gametophytic and sporophyte selection mostly offset in reducing $K_{a}/K_{s}$ when the population size is large.

Both gametophytic and sporophyte selection alone can substantially reduce $\pi_{a}$ as the genetic drift effects become small (fig. 7*B*). The antagonistic selection between two phases facilitates maintenance of higher polymorphisms, compared with the case of single-phase selection (fig. 7*B*).

Under synergistic selection, the mutant allele is deleterious in both phases. A large population size facilitates purging of deleterious alleles in both phases, resulting in decreasing patterns of $K_{a}/K_{s}$ and $\pi_{a}$ as the genetic drift effects become small (supplementary fig. S5, Supplementary Material online). Unlike the case of antagonistic selection, cumulative effects of purging deleterious alleles are present for genes under biphasic selection, which further reduces $K_{a}/K_{s}$ and $\pi_{a}$ (supplementary fig. S5, Supplementary Material online).

## Discussion

The ratio of nonsynonymous to synonymous divergence at a nucleotide site, $K_{a}/K_{s}$, is widely applied to detecting natural selection with molecular sequence data. To estimate $K_{a}/K_{s}$, we may use pairwise or multiple homologous (mostly orthologous but less frequently paralogous) protein-coding gene sequences. With the pairwise sequences, we count the numbers of synonymous and nonsynonymous sites in each sequence and their differences at synonymous and nonsynonymous sites. We then calculate the proportions of synonymous and nonsynonymous differences. The evolutionary rate per nucleotide site is estimated according to a specific model of molecular evolution, such as the one-parameter model (Jukes and Cantor 1969). With multiple gene sequences, the maximum likelihood estimates of $K_{a}/K_{s}$ at a nucleotide site can be obtained using phylogeny-based method (Yang 2006). For instance, the CODEML program from PAML (Yang 2007) or MEGA package (Kumar et al. 2018) can be applied to estimating this ratio. Although it is well known that $K_{a}/K_{s}$ varies across sites along a gene sequence, such a “spatial” variation across sites with positive and purifying selection partly offsets and makes it difficult to detect selection at the gene level. Here, we demonstrate that phase variation of selection during the life cycle may also make it difficult to detect selection for genes co-expressed across phases. Selfing can regulate $K_{a}/K_{s}$ through its interaction with biphasic selection. In addition, we show that $\pi_{a}$ or $\pi_{a}/\pi_{s}$ also exhibits different patterns under one-phasic or biphasic selection. All these results aid in characterizing molecular evolution under phase variation of selection and diverse systems of mating.

### Specific Conclusions

Several specific conclusions can be drawn below:

1. Opposite effects occur in antagonistic selection between gametophytic and sporophytic phases, leading to compensation in estimating $K_{a}/K_{s}$ for mutant alleles under biphasic selection. However, cumulative effects on $K_{a}/K_{s}$ occur for mutant alleles under synergistic biphasic selection. Under the additive selection model (*h* = 1/2) and comparable strength of biphasic allelic selection ($s_{d}=2s_{h}$), gametophytic selection is less effective than sporophytic selection in changing $K_{a}/K_{s}$ in the mixed or selfing mating system (0 < *α* ≤ 1) but equally effective in the outcrossing system (α = 0).
2. Selfing oppositely interacts with gametophytic and sporophytic selection in shaping $K_{a}/K_{s}$ and $\pi_{a}$ or $\pi_{a}/\pi_{s}$ in either antagonistic or synergistic selection. Under the additive selection model, selfing can increase or reduce $K_{a}/K_{s}$, depending upon the relative selection strength of the mutant allele in two phases. Selfing reduces efficacy of gametophytic selection. Under the nonadditive selection model (h≠1/2), partial dominance (h>1/2) strengthens the purging of deleterious alleles in the sporophyte phase, while partial recessivity (h<1/2) facilitates masking deleterious alleles in heterozygotes and weakens the purging of deleterious alleles.
3. Gene flow reduces $K_{a}/K_{s}$ of the mutant allele in both antagonistic and synergistic selection. Gene flow reduces selection efficacy but may increase $\pi_{a}$ or $\pi_{a}/\pi_{s}$. Seed flow has larger effects than pollen flow on $K_{a}/K_{s}$ and $\pi_{a}$ or $\pi_{a}/\pi_{s}$, given their same migration rates.
4. Genetic drift has opposite effects on $K_{a}/K_{s}$ for genes expressed in one or two phases under antagonistic selection, depending on the relative selection coefficients and the type of mating system. However, genetic drift has the same directional effects on $K_{a}/K_{s}$ for genes expressed in two phases under synergistic selection.

### Model Assumption and Comparison

Previous studies have examined the properties of $K_{a}/K_{s}$ and $\pi_{a}/\pi_{s}$ by emphasizing the effects of selfing on the effective population size and genetic hitchhiking (Glemin 2007; Glemin and Muyle 2014). They showed that the reduction in effective population size can relax selection against deleterious alleles (Glemin et al. 2019; Li et al. 2023). They confirmed that difference between the two ratios is pronounced between selfers and outcrossers at equilibrium. Here, we also study the effects of selfing on the effective population size but exclude genetic hitchhiking or background selection effects (Maynard-Smith and Haig 1974; Charlesworth et al. 1993). Besides, we consider phase variation of selection in a life cycle and separation of gene flow via pollen and seeds. The multiple-site–based method was used by Glemin (2007), where three probabilities of nonsynonymous mutations (neutral, deleterious, and advantageous) were jointly considered according to the infinite-site mutation model. The present analysis is individual site based, assuming that the nonsynonymous site is under weak selection (Welch et al. 2008) while the synonymous site is neutral.

The site model is appropriate for interpreting the results derived from individual site analyses, such as the site model presented by Yang (2007). Two alleles are considered at a single site because more than two alleles at a nucleotide site are infrequent in natural populations. Fixation of a mutant allele is governed by selection, drift, and migration processes. When the mutation effects are included, a more complicated fixation probability is needed to derive, such as the theory of mutational influx equilibrium (Wright 1938; Sawyer and Hartl 1992) and the nonequilibrium theory of site-frequency spectrum (Evans et al. 2007). The present single-site model differs from the infinite-site model on which site-frequency spectrum theory is based. Polymorphisms can be maintained at synonymous sites under mutational influx ($\pi_{s}\neq 0$). Also, inclusion of mutational influx in a single-site model could likely yield a comparable $\pi_{a}/\pi_{s}$ ratio to that derived from site-frequency spectrum theory in an isolated population ($\tilde{m}$ = 0; Sawyer and Hartl 1992). This needs further clarifications. If the continuous selection effects of mutants are considered, such as a gamma distribution (Piganeau and Eyre-Walker 2003), an appropriate extension is required (Kryazhimskiy and Plotkin 2008).

The theory assumes that sites are independent from each other. When multiple sites are considered simultaneously, linkage disequilibria (LD) between sites could be generated by selfing through reducing recombination rate (Glemin 2007) or by seed and pollen flow. Although analytical fixation probability for a mutant allele is unavailable under LD, it is speculated that fixation probability of a mutant allele at a nonsynonymous site could be influenced by its linked selective sites (Maynard-Smith and Haig 1974; Charlesworth et al. 1993). Positive (or negative) LD between a mutant allele and its linked background adaptive alleles facilitate (or impede) its fixation. Similarly, fixation of a mutant neutral allele could be facilitated by its closely linked adaptive allele or impeded by its linked deleterious alleles. $K_{a}/K_{s}$ and $\pi_{a}$ or $\pi_{a}/\pi_{s}$ are also influenced by LD. Thus, a caution is needed in interpreting the observed evolutionary rate at nucleotide sites. The present theory confines to the single site–based analysis.

In a relevant theory of antagonistic selection, Peters and Weis (2018) also showed that selfing is critical in spreading and maintaining pollen-expressed genes related to competitiveness. Selfing impedes the fixation of the alleles conferring greater pollen competitiveness. Here, besides the pollen selection (competition), allele selection in ovules is considered because ovule selection could not be excluded in reality (Hu and Li 2003). One restrictive assumption is that only the same fitness is allowed for an allele between pollen and ovules. This could be violated for those genes that are differentially expressed in male and female gametophytes. For gametophyte specific genes, the present model only confines to those genes comparably expressed across male and female gametophytes.

Concerning the effects of gene flow on $K_{a}/K_{s}$ and $\pi_{a}/\pi_{s}$, the unidirectional migration from mainland to island population reduces selection efficacy but aids in maintaining polymorphisms. The precondition of this function is the presence of a higher level of polymorphisms in mainland population than in recipient population. The impacts of pollen flow are counteracted by mating system through the discounting of alien pollen, different from seed flow. The same conclusion about effects of gene flow on $K_{a}/K_{s}$ is also reported in a model of subdivided populations (Glemin 2007), except that gene flow is not separated into pollen and seed flow.

Our model only considers weak selection and the same order of gene flow and genetic drift effects. If selection is strong, the advantageous allele would be rapidly fixed in one phasic selection alone or in biphasic synergistic selection. Strong selection facilitates fixation of alternative alleles in different species and yields a large $K_{a}/K_{s}$ ratio (>1) in one species. However, even in the presence of strong selection, antagonistic selection contributes to compensating $K_{a}/K_{s}$ and keeping it to approach 1. If migration is high, selection efficacy could be substantially reduced if the allele frequencies are different between migrants and the recipient population (Hu et al. 2003).

### Implications

Several implications can be derived from the present theory. The first implication concerns the detection of natural selection through comparing $K_{a}/K_{s}$ estimates with $K_{a}/K_{s}$ = 1 (neutrality) for the genes expressed in two phases. Although antagonistic selection enhances maintenance of allelic polymorphism (Haldane 1932; Damgaard et al. 1994; Peters and Weis 2018), the opposite effects of biphasic selection weaken the power of detecting selection. This is analogous to the opposite effects of positive and purifying selection across sites along a sequence, which weakens the power of detecting selection at the gene level (Yang 2006). The phase variation of selection in plant life cycles is neglected in our current analysis in terms of $K_{a}/K_{s}$. In addition, selfing reduces the efficacy of gametophytic selection or reduces the cumulated effects of gametophytic and sporophytic selection in the nonadditive (h≠1/2) synergistic scenario. This also weakens the statistical test of natural selection in terms of $K_{a}/K_{s}$.

The second implication concerns the sampling strategy for studying molecular evolution. Both evolutionary rate and selection strength in terms of $K_{a}/K_{s}$ could be different between gametophytic and sporophytic phases (Szovenyi et al. 2013; Immler 2019). Samples collected from the sporophyte phase (e.g., tree leaves) or diploid-expressed genes contain the compound effects from both gametophytic and sporophytic selection, which is difficult to separate. Samples collected from the gametophyte phase (e.g., pollen grains or ovules) or haploid-expressed genes can be used to assess the strength of gametophytic selection (Szovenyi et al. 2013). Separation of gametophytic from sporophytic selection remains a challenge when studying genes with pleiotropic effects in both phases.

The third implication is the theoretical application to interpreting empirical findings of distinct evolutionary rates for genes expressed in alternative generations. The significance of gametophytic selection is previously appreciated (Ottaviano and Mulcahy 1989; Charlesworth and Charlesworth 1992) but recently re-emphasized (Immler and Otto 2018; Beaudry et al. 2020). Gametophytic selection could potentially affect the evolution in predominantly haploid organisms (Immler 2019). For instance, pollen-specific genes had stronger purifying or positive selection than sporophytic-specific genes in highly outcrossing species *Capsella grandiflora* (Arunkumar et al. 2013), but a reverse pattern was observed in *Macrocystis pyrifera* (Molano et al. 2022). The present theory predicts that the evolutionary rate of gametophyte-specific genes is shaped by the selfing rate and exhibits different patterns in selfers and ourcrossers. Empirical evidence already supports this prediction. For instance, Szovenyi et al. (2013) inferred that selection for haploid-specific genes was not more efficient in the haploid stage than for the diploid-specific genes in the diploid stage in the selfing species *Arabidopsis thaliana* (Page and Grossniklaus 2002) and the haploid selfing moss *Funaria hygrometrica* (Szovenyi et al. 2014). This is also evident in a separate study in *A. thaliana*, *Oryza sativa*, *Glycine max*, and in *Arabis alpina* (Gossmann et al. 2016; Gutierrez-Valencia et al. 2021). More empirical studies are needed to verify this prediction.

For genes co-expressed in haploid and diploid phases, empirical results show that they evolve more slowly than one phase-specific gene (Park and Choi 2010; Szovenyi et al. 2013). When a gene has substantially differential expression in two phases, it is likely related to different selection pressures between phases. Based on our theory, antagonistic selection is potentially involved because synergistic selection yields a faster overall evolutionary rate for the biphase-expressed genes than for one phase-specific genes. Other types of selection schemes cannot be excluded.

Finally, the theory helps to infer the mode of selection (antagonistic vs. synergistic) by combining the patterns of $K_{a}/K_{s}$ and $\pi_{a}$ or $\pi_{a}/\pi_{s}$ and the type of mating system. For genes with pleiotropic effects in haploid and diploid phases, there are substantial differences in synergistic selection but small differences in antagonistic selection between selfing and outcrossing species in the $K_{a}/K_{s}$ and $\pi_{a}$ or $\pi_{a}/\pi_{s}$ ratios. For genes expressed in the sporophytic phase, similar patterns of $K_{a}/K_{s}$ and $\pi_{a}$ or $\pi_{a}/\pi_{s}$ are expected between selfing and outcrossing species. For genes expressed in the gametophytic phase only, substantial differences are expected between selfing and outcrossing species in $K_{a}/K_{s}$ and $\pi_{a}$ or $\pi_{a}/\pi_{s}$. These predictions need verifications in future studies.

The relative strength of gametic versus zygotic selection plays an important role in shaping $K_{a}/K_{s}$ for genes with pleiotropic effects. Practical observations of high $K_{a}/K_{s}$ ratios in species with mixed mating systems could imply strong positive selection in the sporophytic phase (Wang et al. 2021; Li et al. 2023). This likely occurs in most plants that have a much longer sporophyte phase than gametophyte phase, such as forest trees. Long-lived species could have more chances of undergoing stronger selection pressure in the sporophyte phase than that in the gametophyte phase, in contrast to short-lived species. Under this situation, sporophytic selection is likely stronger than gametophytic selection (Hu et al. 2019), and gametophytic selection could be negligible. Otto and Marks (1996) showed that, in theory, selfing or inbreeding favors the gametophyte phase, while outcrossing favors the sporophyte phase. If $K_{a}/K_{s}$ is low in selfing/inbreeding species, purifying gametophytic selection could be strong, and vice versa. If $K_{a}/K_{s}$ is high in predominantly outcrossing species, gametophytic selection could also be strong, and vice versa. These predictions await appropriate data collection.

## Supplementary Material

evad151_Supplementary_Data

## Data Availability

The Supplementary material includes supplementary appendices S1 and S2, table S1, and figures S1–S5, Supplementary Material online. Two Mathematica notebooks for numerical calculations are provided in separate files.
