# Supplementary material for: The Ka /Ks and πa /πs Ratios under Different Models of Gametophytic and Sporophytic Selection
Source: Genome Biol Evol. 2023 Aug 10;15(8):evad151. doi: 10.1093/gbe/evad151 (PMC10443736; doi:10.1093/gbe/evad151)
Supplement: evad151_Supplementary_Data [file evad151_supplementary_data.zip › Supporting Information_202308.docx]

**The *K_a_/K_s_* and *π_a_/π_s_* ratios under different models of gametophytic and sporophytic selection**

Ling-Ling Li^1,2^, Yu Xiao^1,2^, Xi Wang^1,2^, Zi-Han He ^1,2^, Yan-Wen Lv ^1,2^, Xin-Sheng Hu^1,2*^

1. College of Forestry and Landscape Architecture, South China Agricultural University, Guangzhou 510642, China

2. Guangdong Key Laboratory for Innovative Development and Utilization of Forest Plant Germplasm, Guangzhou 510642, China

* Correspondence**:** Xin-Sheng Hu, Email: [xinsheng@scau.edu.cn](mailto:xinsheng@scau.edu.cn)

**Running title**: *K_a_/K_s_* and *π_a_/π_s_* under biphasic selection

**Supporting Information**

**Appendix A: Recursive expression of gene frequency**

Here we derive the recursive expression for gene and genotype frequencies at one locus with two alleles (*A*, *a*) in the island population under the mainland-island model. We only consider unidirectional gene flow (pollen and seed flow) from mainland to island population. Assume that the mainland population is stable in genetic composition. The derivations are based on the life cycle of a hermaphrodite plant species mentioned in Figure 1 in the main text, including generation of pollen and ovules, pollen flow, selection at the gametophyte phase, mixed mating, seed flow, mutation, selection at the sporophyte phase and genetic drift. In deriving the gene and genotypic frequencies under a mixed mating system, we follow Wright’s (1969) idea where the selfing and random mating parts are separately considered and then combined before seed flow.

Let $p_{AA}$, $p_{Aa}$ and $p_{aa}$ be the frequencies of genotypes *AA*, *Aa* and *aa* in adults in the island population at generation $t$. Let $F$ be the inbreeding coefficient. The genotypic frequencies in adults can be generally expressed as $p_{AA}=p_{A}^{2}+p_{A}p_{a}F$, $p_{Aa}=2p_{A}p_{a}(1-F)$ and $p_{aa}=p_{a}^{2}+p_{A}p_{a}F$. Let α the selfing rate, and 1-α is the outcrossing rate. First, consider the selfing part. At the gametophyte phase, let the fitness of alleles $A$ and $a$ be $w_{A}=1-s_{h}$ and $w_{a}=$1, respectively. Gametophytic selection does not occur in homozygotes *AA* and *aa*, but occur in heterozygote *Aa*. The average fitness in pollen $\bar{w}_{P}$ and ovules $\bar{w}_{O}$ in heterozygote *Aa* is $\bar{w}_{P}$=$\bar{w}_{O}$=$1-\frac{1}{2}s_{h}$. Thus, the genotypic frequencies in seeds after gametophyte selection are summarized as follows:

$p_{AA.S}=p_{AA}+\left( \frac{1-s_{h}}{2-s_{h}} \right)^{2}p_{Aa}$

=$p_{A}^{2}+\frac{1}{2}p_{A}p_{a}\left( 1-s_{h} \right)+Fp_{A}p_{a}\left( 1-\frac{1}{2}\left( 1-s_{h} \right) \right)$ (A1)

$p_{Aa.S}=\frac{2(1-s_{h})}{\left( 2-s_{h} \right)^{2}}p_{Aa}$

=$p_{A}p_{a}(1-F)$ (A2)

$p_{aa.S}=p_{aa}+\frac{1}{\left( 2-s_{h} \right)^{2}}p_{Aa}$

=$p_{a}^{2}+\frac{1}{2}p_{A}p_{a}\left( 1+s_{h} \right)+Fp_{A}p_{a}\left( 1-\frac{1}{2}\left( 1+s_{h} \right) \right)$ (A3)

Next, consider the outcrossing part. The allele frequencies in pollen or in ovules before the occurrence of pollen flow remain $p_{A}$ and $p_{a}$ ($p_{A}$+$p_{a}$=1). After pollen flow, let $p_{A.P}^{*}$ and $p_{a.P}^{*}$ be the frequencies of gametes *A* and *a* in pollen, respectively. The allele frequency in ovules remains unaltered. The gametic frequency in pollen is expressed as:

$p_{A.P}^{*}=m_{P}Q_{A}+\left( 1-m_{P} \right)p_{A}$ (A4)

where $Q_{A}$ is the allele frequency in migrants of pollen grains, which is the same as the allele frequency in the mainland population. The gametic frequency $p_{a.P}^{*}$ can be expressed in the way similar to (A4).

Consider the selection in the gametophyte phase. The average fitness in pollen is

$\bar{w}_{P}=1-s_{h}p_{A.P}^{*}=1-s_{h}p_{A}+O(s_{h}m_{P})$ (A5)

We only consider weak selection so that the terms with the product of $s_{h}m_{P}$ are neglected. The average fitness in ovules is

$\bar{w}_{O}=1-s_{h}p_{A}$ (A6)

which is equal to $\bar{w}_{P}$. Thus, the allele frequency in pollen and ovules after gametophytic selection is derived as:

$p_{A.P}^{**}=p_{A.P}^{*}-p_{A}p_{a}s_{h}$ (A7)

$p_{A.O}^{*}=p_{A}-p_{A}p_{a}s_{h}$ (A8)

The frequency of allele *a* is $p_{a.P}^{**}=1-p_{A.P}^{**}$ in pollen and $p_{a.O}^{*}=1-p_{A.O}^{*}$ in ovules.

After random combination between pollen and ovules, the genotypic frequencies in seeds so formed are derived as

$p_{AA.R}=p_{A}p_{A.P}^{*}-2p_{A}^{2}p_{a}s_{h}+O(s_{h}^{2}, s_{h}m_{P})$ (A9)

$p_{Aa.R}=p_{A.P}^{*}p_{a}+p_{a.P}^{*}p_{A}+2p_{A}p_{a}\left( p_{A}-p_{a} \right)+O(s_{h}^{2}, s_{h}m_{P})$ (A10)

$p_{aa.R}=p_{a.P}^{*}p_{a}+2p_{A}p_{a}^{2}s_{h}+O(s_{h}^{2}, s_{h}m_{P})$ (A11)

The genotypic frequencies after the mixed mating system are derived by combining the selfing and outcrossing parts and are further simplified as:

$p_{AA}^{*}=\alpha p_{AA.S}+(1-\alpha)p_{AA.R}$

=$\alpha p_{A}\left( p_{A}+Fp_{a}+\frac{1}{2}p_{a}(1-s_{h})(1-F) \right)$

$+(1-\alpha)p_{A}\left( m_{P}Q_{A}+\left( 1-m_{P} \right)p_{A}-2p_{A}p_{a}s_{h} \right)$ (A12)

$p_{Aa}^{*}=\alpha p_{A}p_{a}(1-F)$

$+(1-\alpha)\left( m_{P}\left( Q_{A}p_{a}+Q_{a}p_{A} \right)+2\left( 1-m_{P} \right)p_{A}p_{a}+2p_{A}p_{a}\left( p_{A}-p_{a} \right)s_{h} \right)$ (A13)

$p_{aa}^{*}=\alpha p_{aa.S}+(1-\alpha)p_{aa.R}$

=$\alpha p_{a}\left( p_{a}+Fp_{A}+\frac{1}{2}p_{a}(1+s_{h})(1-F) \right)$

$+(1-\alpha)p_{a}\left( m_{P}Q_{a}+\left( 1-m_{P} \right)p_{a}+2p_{A}p_{a}s_{h} \right)$ (A14)

After seed flow from the mainland to island population, the genotypic frequencies are simply given by

$p_{AA}^{**}=m_{S}Q_{AA}+(1-m_{S})p_{AA}^{*}$ (A15)

$p_{Aa}^{**}=m_{S}Q_{Aa}+(1-m_{S})p_{Aa}^{*}$ (A16)

$p_{aa}^{**}=m_{S}Q_{aa}+(1-m_{S})p_{aa}^{*}$ (A17)

$Q_{AA}$, $Q_{Aa}$ and $Q_{aa}$ are the genotypic frequencies in migrants of seeds, the same as those in the mainland population.

Now we consider selection in the sporophyte phase. Let the fitness of three genotypes be $W_{AA}=1$ , $W_{Aa}=1-hs_{d}$, and $W_{aa}=1-s_{d}$. The average fitness in the sporophyte phase is simplified as $\bar{W}=W_{AA}p_{AA}^{**}+W_{Aa}p_{Aa}^{**}+W_{aa}p_{aa}^{**}$, which is simplified as

$\bar{W}=1-hs_{d}p_{A}p_{a}\left( 2-\alpha\left( 1+F \right) \right)-s_{d}p_{a}\left( p_{a}+\frac{1}{2}\alpha p_{A}(1+F) \right)$ (A18)

After selection, the genotypic frequencies are

$p_{AA}^{***}=\frac{p_{AA}^{**}}{\bar{W}}$

= $p_{AA}^{**}+p_{AA}^{**}\left( 1-\bar{W} \right)$ (A19)

$p_{Aa}^{***}=\frac{{(1-hs_{d})p}_{Aa}^{**}}{\bar{W}}$

= $p_{Aa}^{**}-hs_{d}p_{Aa}^{**}+p_{Aa}^{**}\left( 1-\bar{W} \right)$ (A20)

$p_{aa}^{***}=\frac{{(1-s_{d})p}_{aa}^{**}}{\bar{W}}$

= $p_{aa}^{**}-s_{d}p_{aa}^{**}+p_{aa}^{**}\left( 1-\bar{W} \right)$ (A21)

After sophisticated calculations in algebra, the gene frequency in the next adults before genetic drift is derived as

$p_{A}^{***}=p_{AA}^{***}+\frac{1}{2}p_{Aa}^{***}$

= $p_{A}+\left( m_{S}+\frac{1-\alpha}{2}m_{P} \right)\left( Q_{A}-p_{A} \right)$

$-s_{h}p_{A}p_{a}\left( 1-\frac{1}{2}\alpha(1+F) \right)$

$+s_{d}p_{A}p_{a}\left( p_{a}+\frac{1}{2}\alpha p_{A}\left( 1+F \right)+h\left( p_{A}-p_{a} \right)\left( 1-\frac{1}{2}\alpha(1+F) \right) \right)$ (A22)

This recursive expression is then used to calculate the systematic change of gene frequency.

**Appendix B: Proof of a positive** $\boldsymbol{f(\epsilon,q)}$ **function**

Let $\epsilon=2N(s_{d}-2\left( 1-\alpha\right)s_{h})$ where *N* is the actual population size, $s_{d}$ and $s_{h}$are the selection coefficients at gametophyte and sporophyte phases respectively, and $\alpha$ is the selfing rate (0$\leq\alpha\leq1$). Let *q* be the initial allele frequency. Consider the following function:

$f\left( \epsilon,q \right)=qe^{\epsilon}-q-e^{\epsilon q}+1$ (B1)

If $\epsilon$ is a positive value, use of the Taylor series expansion yields

$f\left( \epsilon,q \right)=q\left( 1+\epsilon+\frac{\epsilon^{2}}{2!}+\frac{\epsilon^{3}}{3!}+\ldots\right)-q-\left( 1+\epsilon q+\frac{\left( \epsilon q \right)^{2}}{2!}+\frac{\left( \epsilon q \right)^{3}}{3!}+\ldots\right)+1$

$=\frac{\epsilon^{2}}{2!}q\left( 1-q \right)+\frac{\epsilon^{3}}{3!}q\left( 1-q^{2} \right)+\ldots+\frac{\epsilon^{n}}{n!}q(1-q^{n-1})$ (B2)

Since allele frequency $q$ is smaller than 1, $f(\epsilon,q)$ is then greater than zero.

If $\varepsilon<0$, let $\epsilon=-\xi$ and $\xi>0$. The function $f\left( \epsilon,q \right)$ is re-expressed as $f\left( \epsilon,q \right)=f\left( -\xi,q \right)$. Using the Taylor series expansion, we derived

$f\left( -\xi,q \right)=e^{-\xi(1+q)}\left( qe^{\xi q}-qe^{\xi\left( 1+q \right)}-e^{\xi}+e^{\xi(1+q)} \right)$

$=e^{-\xi(1+q)}\left( \xi\left( 1-q \right)+\frac{\xi^{2}}{2!}\left( \left( 1+q \right)^{2}\left( 1-q \right)-1+q^{3} \right)+\ldots\right.$

$+\left. \frac{\xi^{n}}{n!}\left( \left( 1+q \right)^{n}\left( 1-q \right)-1+q^{n+1} \right) \right)$ (B3)

Thus, $f\left( -\xi,q \right)$ is positive.

**Table S1**. Symbols and biological meanings used in the theory

| Symbol | Biological meaning |
| --- | --- |
| $K_{a}$ | The substitution rate of nucleotides at a nonsynonymous site |
| $K_{s}$ | The substitution rate of nucleotides at a synonymous site |
| *K_a_/K_s_* | The ratio of nonsynonymous to synonymous divergence per site |
| *π_a_/π_s_* | The ratio of nonsynonymous to synonymous polymorphism per site |
| $s_{h}$ | Selection coefficient in the gametophyte phase; |
| $s_{d}$ | Selection coefficient in the sporophyte phase |
| $h$ | Degree of dominance |
| $W_{AA}$, $W_{Aa}$, $W_{aa}$ | Fitnesses of genotypes *AA*, *Aa* and *aa*, respectively |
| $p_{AA}$, $p_{Aa}$, $p_{aa}$ | Frequencies of genotypes *AA*, *Aa* and *aa*, respectively |
| $w_{A}$, $w_{a}$ | Fitnesses of alleles *A* and *a*, respectively |
| $w_{P}$, $w_{O}$ | Mean fitnesses in pollen and ovules, respectively |
| *α* | Selfing rate |
| $p_{i}^{*}$($i=A,a$) | Allele frequency after pollen flow |
| $p_{i}(i=A,a)$ | Allele frequency at current adults |
| $\bar{W}$ | Mean fitness in the sporophyte phase |
| $p_{ij}^{**}$ | Frequency of genotype *ij* after seed flow |
| $F$ | Inbreeding coefficient |
| $p_{A}^{***}$ | Frequency of allele *A* after selection in the sporophyte phase |
| $m_{S}$, $m_{P}$ | Migration rates of seeds and pollen, respectively |
| $\tilde{m}$ | Composite migration rate (=$m_{S}+\left( 1-\alpha\right)m_{P}/2$) |
| $M_{\Delta p_{A}}$, $M_{\Delta p_{a}}$ | Per generation systematic changes of allele frequencies for *A* and *a* , respectively |
| $V_{\Delta p_{A}}$, $V_{\Delta p_{a}}$ | Variances for the per generation change of gene frequency $\Delta p_{A}$ and $\Delta p_{a}$, respectively |
| $u$ | Mutation rate from allele *A* to *a* |
| $\mu$ | Mutation rate at the synonymous site |
| $Q_{A}$, $Q_{a}$ | Migrant allele frequencies of *A* and *a* from mainland population, respectively |
| $N$ | Actual population size |
| $N_{e}$ | Effective population size |
| $\varphi\left( p_{0} \right)$ | Fixation probability of a mutant allele with initial frequency $p_{0}$ |
| $\varphi_{0}\left( p_{0} \right)$ | Fixation probability of a neutral allele with initial frequency $p_{0}$ |
| $G\left( p_{a} \right)$ | Function of allele frequencies $p_{a}$ |
| $\phi\left( p_{a} \right)$ | Density function of allele frequency $p_{a}$ |
| $\bar{p}_{A}$, $\bar{p}_{a}$ | Expected frequencies of alleles *A* and *a* at equilibrium, respectively |
| $V_{p}$ | Variance of allele frequency at equilibrium |

**Figures S1-S5**

**Figure S1**. Examples of selfing effects on ${K_{a}}/{K_{s}}$ and $\pi_{a}$ under synergistic selection. Results in (A) are derived from equation (20) and in (B) from a Mathematica notebook. The parameters except for selection coefficients shown in figures are the population size $N$=30, the degree of dominance $h$=0.5, the initial allele frequency $p_{0}=1/2N$, and the migration rates of seeds and pollen $m_{S}=m_{P}=0$.

**Figure S2**. Examples of the effects of seed flow on ${K_{a}}/{K_{s}}$ and $\pi_{a}$ or ${\pi_{a}}/{\pi_{s}}$ under antagonistic selection in a predominantly outcrossing system (α=5%). Results are derived from a Mathematica notebook. Parameters except for those shown in figures are the population size *N*=30, the initial allele frequencies $p_{0}=1/2N,$the degree of dominance *h*=0.5, the migration rate of pollen $m_{P}=0$, and the migrant allele frequencies $Q_{A}=Q_{a}$=0.5.

**Figure S3**. Examples of the effects of pollen flow on ${K_{a}}/{K_{s}}$ and $\pi_{a}$ or ${\pi_{a}}/{\pi_{s}}$ under synergistic selection in a predominantly outcrossing system (α=5%). Results are derived from a Mathematica notebook with following parameters: the degree of dominance $h=0.5$, population size *N*=30, initial allele frequency $p_{0}=1/2N$, the migration rate of pollen $m_{S}=0$, and the migrant allele frequencies $Q_{A}=Q_{a}$=0.

**Figure S4**. Examples of the effects of seed flow on $\pi_{a}$ or ${\pi_{a}}/{\pi_{s}}$ under synergistic selection in a predominantly outcrossing system (α=5%). Results are derived from a Mathematica notebook. Parameters except for those shown in figures are the population size *N*=30, the initial allele frequencies $p_{0}=1/2N,$the degree of dominance *h*=0.5, the migration rate of pollen $m_{P}=0$, and the migrant allele frequencies $Q_{A}=Q_{a}$=0.5.

**Figure S5**. Examples of genetic drift effects ${K_{a}}/{K_{s}}$ and $\pi_{a}$ under synergistic selection in a predominantly outcrossing system (α=5%): (A) ${K_{a}}/{K_{s}}$ and (B) $\pi_{a}$. Results are derived from a Mathematica notebook. Parameters except for selection coefficients and population size shown in figures are the initial allele frequencies $p_{0}=1/2N,$the degree of dominance *h*=0.5, and the migration rates of pollen and seeds $m_{P}=m_{S}=0$.
